# The characteristics of Grave’s disease in children and adolescent patients in Al-Madinah Al-Munawwarah: A retrospective chart review

**DOI:** 10.15537/smj.2023.44.3.20220501

**Published:** 2023-03

**Authors:** Rasha S. Alradadi, Noura A. Omar, Renad A. Alhusayni, Amirah M. Almokhtar, Shahad F. Muhawish, Mawaddah M. Altaleb, Sundus A. Tawfiq, Reem M. Alraddadi, Randa Q. Almalki

**Affiliations:** *From the Department of Pediatric Endocrinology, Taibah University, Al-Medinah Al-Munawarah, Kingdom of Saudi Arabia.*

**Keywords:** Graves’ disease, Hyperthyroidism, Children, Adolescents

## Abstract

**Objectives:**

To determine the characteristics of Graves’ disease in children and adolescents in Medina, the Kingdom of Saudi Arabia, and compare them to those observed in other countries.

**Methods:**

This is a retrospective chart review of children and adolescents diagnosed with Graves’ disease between January 2010 and May 2021.

**Results:**

Fifty-eight patients aged 12.02 ± 4.85 years were identified, of which 44 (75.9%) were females. Exophthalmos (63.8%), neck swelling (60.3%), palpitations (46.6%), and tremors (29.3%) were the most common manifestations. Vitiligo (1.72%) and alopecia (1.72%) were the only autoimmune diseases observed in our patients. The median (IQR) value was 0.01 (0.36) (ulU/mL) for thyroid-stimulating hormone (TSH) and 24.89 (29.50) (pmol/L) for FT4. In terms of treatment modalities, 55 (94.8%) received antithyroid medication, 6 (10.3%) underwent thyroidectomy and one patient (1.72%) was treated with radioactive iodine.

**Conclusion:**

In general, Graves’ disease is more common in females. Neck swelling, palpitation, and tremors were the main manifestations. Compared with other countries, there was a higher frequency of exophthalmos and a lower frequency of associated autoimmune conditions. The primary treatment was antithyroid drugs; thyroidectomy and radioactive iodine were used less frequently.

**G**raves’ disease (GD) is the most common cause of hyperthyroidism in children.^1^ It is an autoimmune disease caused by the stimulation of thyroid receptor antibodies (TRAb) or thyroid-stimulating immunoglobulins (TSI). This condition may occur due to both genetic and environmental factors.^2,3^ Worldwide, GD occurs in approximately 0.02% of children (1:5000), with a peak incidence in the age group of 11–15 years and a female-to-male ratio of 5:1.^4^ However, its characteristics and prevalence in the paediatric population are not well known in the Kingdom of Saudi Arabia (KSA). In general, GD can be diagnosed based on the presence of associated symptoms, the occurrence of goitre, and confirmatory blood test findings.^5^ The initial clinical presentation is often severe, especially if the child is young, pre-pubescent, and/or non-Caucasian. Prior research reports the presence of different symptoms globally, such as goitre (100%) and exophthalmia (89%) in Africa, goitre (68.4%) and excessive sweating (53.4%) in Asia, and cardiac symptoms, such as palpitation and tachycardia in the Middle East.^1,6,7^ A local study reported the case of a 4-year-old girl in KSA, who primarily experienced neck swelling, tremors, nervousness, protrusion of both eyes, weight loss and sleep disturbances.^1^ While the case report mentioned different modalities of management, there was no information about the most common modality used in paediatrics in KSA. Therefore, as part of this study, we examine the different modalities used by Mirghani et al^8^ in their study, which included 100 patients from a tertiary health centre in KSA, aged 9 (+/- 5) years old, with psychiatric disorders. The common psychiatric disorders were ADHD and anxiety, with hyperthyroidism being observed in 15% of cases.^8^ Because spontaneous resolution is uncommon, the majority of patients with GD need hyperthyroidism treatment. Key therapeutic options for GD include radioactive iodine treatment (I131), surgery, or medical treatment with antithyroid drugs (ATD), propylthiouracil (PTU) or methimazole.^9^

The findings of a study that was conducted on adult patients with GD in KSA revealed certain differences in the characteristics of the disease compared to those reported in other countries.^10^ However, there is insufficient data to fully represent the characteristics of the disease in paediatric and adolescent populations in KSA. Therefore, our study aims to determine the characteristics of GD in children and adolescents in Medina, KSA, and to compare these measures with those reported in other countries.

## Methods

The current study is an observational retrospective study conducted in two major hospitals in Medina, western KSA, namely, the Medina Maternity and Children Hospital (MMCH) and King Fahad Hospital (KFH). The study encompasses the medical records of 58 patients with GD, who were identified either via the computerized hospital databases using the International Classification of Disease (ICD) or by using paper medical records of those who were either hospitalized or who visited the outpatient endocrinology clinic during a 10-year period from January 2010 to May 2021. Ethical approval (IRB 33-2021) was obtained from the appropriate local authority before the study was started. Furthermore, to maintain confidentiality, all patient data were transferred using coding numbers, and no decoding was required at any stage.

The study included patients ≤18 years old who had been diagnosed with GD based on their medical history, clinical manifestations, and laboratory investigation during the specific duration. Patients aged 14–18 years were managed mainly by the adult endocrinology department at KFH. Patients with hyperthyroidism due to other causes, such as Hashimoto’s thyroiditis and toxic thyroid nodule, were excluded.

Among the 58 patients who fit the selection criteria, 44 (75.90%) were female, while only 14 (24.10%) were male. The selected patients’ data were collected directly from their medical records. All relevant information was transferred into a pre-designed data sheet comprised of patient demographics (age, gender, nationality); family history; social history; thyroid function test results; type of treatment, including medications, surgery, and treatment with radioactive iodine; patient compliance and follow-up; and co-morbid conditions.

The laboratory investigations were obtained at the first visit for the diagnosis and were used to follow up on the outcome of the patients.

### Statistical analysis

Data analysis was performed by using Statistical Package for the Social Sciences, version 23.0 (IBM Corp., Armonk, NY, US. The categorical variables are presented as frequencies and percentages. The normally distributed continuous variables are presented as means and standard deviations, while the non-normally distributed data are presented as median and IQR.

The study approved by the Institutional Review Board of the College of Medicine Taibah University, Medina, KSA, KFH, and MMCH.

## Results

Table 1 shows the socio-demographic data of the 58 patients who were included in the study. Fourteen (24.1%) were male, and overall, 42 (72.4%) were of Saudi nationality. The mean (SD) age was 12.02 (4.85) years, with a minimum age of 3 months and a maximum age of 18 years. Eleven patients (19%) had a positive family history of thyroid disease, of which 7 (12.1%) had a family history of GD, one (1.72%) had hyperthyroidism unspecified, 2 (3.5%) had hypothyroidism disease, and one (1.7%) had a family history of thyroid cancer. Regarding associated diseases with Graves’ disease, of the 58 subjects who had Graves’ disease, bronchial asthma was found in 5.2%, and microcytic hypochromic anaemia and epilepsy in 3.5% for each. Psychiatric disease, vitiligo, alopecia, and congenital adrenal hyperplasia were the least common associated diseases 1.7% for each disease. The symptoms and signs of GD are shown in Table 2. The most common symptoms were neck swelling (60.3%) and palpitation (46.6%). Table 3 shows the median and inter-quartile range (IQR) of the laboratory tests performed for the patients prior to diagnosis. Regarding types of treatment, 55 patients (94.8%) received antithyroid medication and 6 (10.3%) had undergone surgery, among whom 5 (8.6%) had undergone total thyroidectomy while one (1.7%) had undergone right hemithyroidectomy. One patient (1.7%) had received radioactive therapy. Table 4 demonstrates the patient status post-treatment. The mean (SD) recovery time was 15.9 (12.9) months with a minimum of one month and a maximum of 48 months.

**Table 1:** - Socio-demographic profile of the participants (n=58).

Demographic characteristics	n	(%)
***Gender***		
Male	14	(24.10)
Female	44	(75.90)
***Nationality***		
Saudi	42	(72.40)
Non-Saudi	16	(27.60)
Age (in years)	12.02 (0.25-18.00)±4.85	
Height	146.38±19.43	
Weight	46.85±21.23	
BMI	20.8±6.02	

**Table 2:** - Patients’ symptoms and signs of hyperthyroidism (Graves’ disease) (N=58).

Symptoms	n	%	Signs	n	%
Neck swelling	35	60.3	Exophthalmos	37	63.8
Palpitation	27	46.6	Palpable goitre	33	60.0
Tremor	17	29.3	Tachycardia	19	32.8
Heat intolerance	13	22.4	Sweating	10	17.2
Irritability	13	22.4	Lid retraction	8	13.8
Weight loss	13	22.4	Altered mental status	4	6.9
Insomnia	11	19.0	Failure to thrive	2	3.4
Diarrhoea	11	19.0			
Nervousness	8	13.8			
Hyperactivity	7	12.1			
Shortness of breath	7	12.1			
Increased appetite	4	6.9			
Decrease in concentration	3	5.2			
Irregular menses/Oligomenorrhea	3	5.2			
Eye symptoms (pain, redness, swelling, double vision)	1	1.7			

**Table 3:** - Laboratory investigations (N=58).

Investigation	Median	IQR
TSH (ulU/mL)	0.010	0.36
FT4 (pmol/L)	24.89	29.50
FT3 (pmol/L)	8.55	12.66
Thyroglobulin (ng/ml)	56.33	275.71
TPO (IU/ML)	600	649.8

IQR - inter-quartile range

**Table 4:** - Patients’ status post-treatment (N=58).

Characteristic	n	(%)
Recovery from hyperthyroid state to euthyroid state*		
Yes	16	27.60
No	11	18.97
***Treatment-Induced Hypothyroidism on Levothyroxine***		
Yes	12	20.70
No	46	79.30
***Laboratory investigations post-treatment median (IQR)***		
TSH (ulU/mL)	9.68 (12.27)	
FT4 (pmol/L)	17.21 (13.12)	
FT3 (pmol/L)	12.38 (16.71)	
***Recovery duration (in months)***		
Mean	15.90	
Standard deviation	12.90	
Minimum	1.00	
Maximum	48.00	

* The remaining patients are lost from follow-up data.

## Discussion

The GD is one of the most common causes of hyperthyroidism and leads to manifestations throughout the body. The present study revealed the characteristics of the patients of this disease to identify any differences when compared to patients from other populations.

The demographic features were similar to findings from previous studies.^6,7,17,18^ The observed female-to-male ratio of 3:1 and mean age of 12.02±4.85 years compared favourably to reported data from other studies, which showed female-to-male ratios ranging from 4:1 to 6:1 and a mean age of 10–15 years.^6,7,9,11-18^

The majority of the patients presented as typical cases of GD; therefore, diagnosis was based on the clinical presentations and biochemical blood test (TSH, FT4, FT3) results. Thyroid antibodies, including TSI and TRAb, were not tested due to the unavailability of these tests. Thyroid scans were performed only in atypical cases to confirm the diagnosis. Based on the physical examinations conducted by expert physicians, none of the goitre cases needed ultrasound. The physicians followed the American Thyroid Association (ATA) guidelines for the diagnosis and management of hyperthyroidism.^23^

The most common presentations of GD in KSA were neck swelling and sympathetic overactivity, including palpitations and tremors. The frequency of exophthalmos in GD was more frequent in children (63.8%) compared to a study performed in European countries (2.7%).^17^ The reason for this apparent disparity has not been explored.

Genetic factors can explain 79% of the pathogenic mechanisms of GD.^6^ The frequency of a family history of thyroid diseases in this study is consistent with a study from France, in which this phenomenon was observed in 15%–20% of cases.^21^ A much higher rate was observed in Japan, with^6^ reporting that 40% of children with GD had a familial history of the disease. Although GD is typically more common in children with other autoimmune conditions,^21^ disorders such as vitiligo and alopecia were observed in only 2 patients (3.4%) in the present study.

In addition to beta-blockers, mainly propranolol, to relieve the thyrotoxic symptoms (56.9%), the most common treatment used to manage GD in this study was ATD, including carbimazole (94.8%). Other studies^1,12,17^ have reported similar first-line treatment. However, a study conducted in Abidjan, Ivory Coast,^18^ demonstrated the benefits of prescribing anxiolytics alongside ATD and beta blockers to treat nervousness and irritability, symptoms that were present in 44% of patients. These symptoms were less common in our study, being present in only 21 patients (36.2%).

In some studies,^13,14^ radioactive iodine therapy (RAI) was preferred as a definitive treatment, in contrast to the medical approach that was largely adopted in this study, where RAI was not a preferred approach of treatment and was performed on only one patient (1.7%) According to data published by the ATA in the United States, only 2% of patients with GD and 7% of patients with GD and thyromegaly have been treated with surgery.^22^ This percentage was higher in our study, in which thyroidectomy was a reason for hospital admission in 6 (10.3%) cases.

### Study limitations

The medical records and data documentation were not advanced in the study institutions, where paper charts were the primary method of documentation until recent years. This created some barriers during data collection, such as missing information and poor documentation. Other limitations included missing follow-up visits. Based on the existing health system, patients were transferred to adult services during their mid- to late-adolescent years, which resulted in the absence of some patients during the transition period. In addition, some blood tests, including those required for thyroid antibodies such as TSI and TRAb, were not available; therefore, the physicians had to depend on the clinical presentation, especially if it was typical for GD. Thyroid scans were performed only in atypical cases.

In conclusion, The characteristics of patients with GD in KSA were similar to those reported in other countries, such as being more common in females, and the major presenting symptoms were very similar. However, there were some differences, such as a higher frequency of exophthalmos and a lower incidence of associated autoimmune conditions. The administration of antithyroid drugs, primarily carbimazole, was the first line of treatment, although a more definitive treatment was still required in some cases. With the advancement of electronic medical records in most institutions in KSA, documentation is expected to improve. This is expected to make similar research more feasible in the future. Moreover, this is likely to result in the data becoming more detailed and comprehensive. In addition, further research is necessary to obtain more information about the characteristics of GD in other areas of Saudi Arabia.
